# Lung sound analysis in infants with risk factors for asthma development

**DOI:** 10.1002/hsr2.379

**Published:** 2021-09-17

**Authors:** Manabu Miyamoto, Shigemi Yoshihara, Hiromi Shioya, Hiromi Tadaki, Tomohiko Imamura, Mayumi Enseki, Hideki Koike, Hiroyuki Furuya, Hiroyuki Mochizuki

**Affiliations:** ^1^ Department of Pediatrics Dokkyo Medical University Mibu Japan; ^2^ Division of Pediatrics National Hospital Organization Yokohama Medical Center Yokohama Japan; ^3^ Department of Pediatrics Tokai University School of Medicine Isehara Japan; ^4^ Department of Basic Clinical Science and Public Health Tokai University School of Medicine Isehara Japan

**Keywords:** acute respiratory infection, asthma, infant, lung sound analysis, risk factor

## Abstract

**Background and objectives:**

Using a lung sound analysis, the prognosis of asthma was investigated in infants with risk factors for asthma development by a long‐term observation.

**Methods:**

A total of 268 infants were included (median age: 8 months old). The lung sound parameters (the ratio of the third and fourth area to the total area under the curve [A_3_/A_T_ and B_4_/A_T_], and the ratio of power and frequency at 50% and 75% of the highest frequency [RPF_50_ and RPF_75_]) were evaluated at the first visit. At 3 years old, using a questionnaire, we examined the relationship between the lung sound parameters and risk factors of asthma development.

**Results:**

Among the 268 infants, 175 infants were in good health and 93 had a history of acute respiratory infection (ARI) within 7 days at the first visit. Among the 3‐ to 12‐month‐old infants with an ARI, the A_3_/A_T_, B_4_/A_T_ values in those with a history of asthma/asthmatic bronchitis, atopic dermatitis, and atopy were smaller than in the infants without such histories. Among the 13‐ to 24‐month‐old infants with an ARI, the A_3_/A_T_ and B_4_/A_T_ values in those with a wheezing history were larger than in the infants without such a history.

**Conclusions:**

The characteristics of the lung sounds in infants with risk factors for asthma development were demonstrated over long‐term follow‐up. Lung sound analyses may be useful for assessing the airway condition of infants.

**Abbreviations**
A_3_
: third area under the curve
ARI: acute respiratory infection
A_T_
: total area under the curve of 100 Hz to the highest frequency of the dB power spectrum
AUC: area under the curve
B_4_
: fourth area under the curve
F_99_
: frequency limiting 99% of the power spectrum
RPF_50_
: ratio of power and frequency at 50% of the highest frequency of the dB power spectrum
RPF_75_
: ratio of power and frequency at 75% of the highest frequency of the dB power spectrum.
Slope: roll‐off from 600 to 1200 Hz

## 1 INTRODUCTION

Early intervention is important for treating childhood asthma.^1^ An atopic condition, family history of allergy, respiratory tract infections, and domestic smoking have been highlighted as risk factors for asthma development.^2^ However, not all children with atopy or recurrent wheezing develop asthma.^3^

It has been recommended to perform an exclusion diagnosis and confirmation of recurrent wheezes, which is the most important symptom of asthma.^1^, ^2^ By combining these risk factors with objective indicators of respiratory physiology, it may be possible to achieve a definitive diagnosis of childhood asthma. However, it is considered difficult to make a definitive diagnosis using objective indicators such as routine lung function tests, as childhood asthma typically has an onset under 5 years of age.^4^, ^5^

The utility of a lung sound analysis as a noninvasive lung function test has been studied.^6^, ^7^ Clinically, current reports have suggested that new breath sound analyses can be used in the clinical evaluation of airway changes.^8^, ^9^, ^10^ The major problems associated with performing lung sound analyses in infants and younger children have gradually been improved.^11^, ^12^ We have assessed the airway condition of infants and children via a lung sound analysis, which is a simple and safe technique,^13^ and the clinical objective information related to lung sound analyses has also been reported.^14^ In addition, we recently made further improvements to the analytical system for lung sound analyses.^15^ Using the new technique, we were able to measure the ratio of the AUC more accurately than in the past.

Regarding the objective evaluation of the airway condition in infants, we herein report our assessment of the relationship between the lung sound parameters and risk factors for asthma development in our multicenter‐participated, prospective, long‐term observation of 3‐year‐old infants.

## 2 SUBJECTS AND METHODS

### 2.1 Study subjects

In an ongoing, multi‐institutional prospective study (Diagnosis of Infantile Asthma Using Lung Sound Analysis; DIAL)^14^, ^16^ conducted from 1 January 2012, to 31 March 2016, a total of 443 infants (mean age at the first visit, 9 months old; range, 3‐24 months old) who attended an infant health checkup at the National Hospital Organization Yokohama Medical Center, Isehara municipal clinic, Yamato Municipal Hospital, Dokkyo University School of Medicine and Uchida Iin Y Child Clinic. All parents agreed to participate in this study.

To target infants in good health, the following exclusion criteria were used: infants with severe diseases of the lung, heart, and other organs and a fever and/or respiratory symptoms. At the first visit, none of the subjects had wheezing on auscultation. According to our previous reports,^16^ infants who had an acute respiratory infection (ARI) within the 7 days prior to their first visit were identified as infants with an ARI. As in a previous study,^16^ since the proportion of subjects with and without an ARI varied significantly with age, we divided the subjects into two age groups. Subjects who were 3 to 24 months old at the first visit were evenly divided into two age groups based on the midline of the age (3‐12 and 13‐24 months old) for a stratified analysis (Table 1).

**TABLE 1:** Characteristics of the infants with and without ARI

	ARI negative^a^	ARI positive	*P* value
【Age at first visit: 3‐12 months】 n = 175			
Number of subjects	142	33	—
Age (months)	36 (36, 38)^b^	36 (36, 37)	0.993
First visit (months)	7 (7, 8)	7 (7, 8)	0.276
Sex (male/female)	72 / 70	22 / 11	0.099
Height (cm)	93.0 (90.0, 96.0)	93.5 (89.3, 97.0)	0.603
Weight (kg)	14.0 (13.0, 15.0)	13.0 (12.0, 14.0)	0.170
【Age at first visit: 13‐24 months】 n = 93			
Number of subjects	45	48	—
Age (months)	36 (36, 36)	36 (36, 36)	0.264
First visit (months)	18 (18, 18)	18 (18, 19)	0.217
Sex (male/female)	20 / 25	22 / 26	0.894
Height (cm)	91.3 (88.8, 93.5)	92.0 (89.9, 94.2)	0.463
Weight (kg)	13.7 (12.6, 15.0)	13.5 (12.4, 14.9)	0.823

^a^ From the DIAL study (Reference 14).

^b^ Median (first quartile, third quartile), *P* value; Mann‐Whitney *U* test.

Written informed consent was obtained from all of the legal guardians, and the study protocol was approved by the institutional review board of Tokai University Hospital (No. 11R‐158, approval date; 21 December 2011, No. 14R‐133 approval date; 15 December 2015, No. 17R‐161 approval date; 10 October 2017).

### 2.2 Study protocol

#### 2.2.1 At the first visit

When each subject took tidal breaths, the lung sounds were collected. It was confirmed that the lung sound data included no wheezing, rales, or outside noises based on auscultation by a physician and the lung sound analyzer image.

#### 2.2.2 At 3 years old

When the subjects were 3 years old, an original Japanese questionnaire based on ATS‐DLD^17^ was mailed (Table A1). In this questionnaire, passive smoking was defined by the presence of family smokers. Domestic pets referred to a cat, dog, or other animals with fur. Positivity for a family history of allergy meant a family member within two degrees of relation had a history of allergic diseases.

### 2.3 Breath sound analyses

All participants underwent the collection of lung sounds as previously described.^9^, ^13^ Lung sounds were recorded for 10 or more breaths by a handheld microphone in a quiet booth. The microphone was placed on the upper right anterior chest at the second intercostal space along the mid‐clavicular line. The sound of the inspiration phase was analyzed by an LSA‐2008 sound spectrometer (Kenz Medico Co., Saitama, Japan).

**FIGURE A1:**
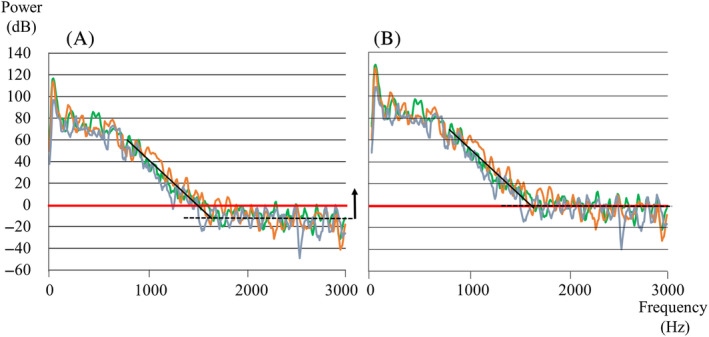
Our new technique for performing a lung sound analysis. The zero level of the original sound spectrum (A, dashed line) was visually corrected based on the background noises of the sound spectrum (B) before the zero point was decided in each sample

To evaluate the dBm‐based spectrum images, we decided to set the zero point of the Y‐axis (dBm) based on the power of background noises in each subject. The zero level (0 dB of lung sound spectrum) was visually corrected based on the lung sound spectra in each sample before the zero point (the frequency at 0 dB) was decided^15^ (Figure A1A,B).

The slope indicates the roll‐off of the middle spectrum curve (−dB/octave).^18^, ^19^ A_T_, A_3_, and B_4_ were conventionally calculated according to the dB and Hz (1 arbitrary unit [dB・Hz] on a spectrum image). The spectrum curve indices (the A_3_/A_T_, B_4_/A_T_, RPF_75_, and RPF_50_) were also calculated. A_3_/A_T_ and B_4_/A_T_ are the numerical values of the ratio of high‐pitched sound area compared with the whole area in the breath sound spectrum, and RPF_75_ and RPF_50_ are the numerical values of the angle of the high‐pitched area in the breath sound spectrum. According to previous reports, the values of parameters increase with bronchial dilatation and decrease with bronchial constriction, and the values of A_3_/A_T_, B_4_/A_T_, RPF_75_, and RPF_50_ also decrease.^11^, ^12^ In addition to the above, the frequency limiting 99% of the power spectrum (F_99_) was also measured in accordance with the methods of previous reports.^18^, ^19^, ^20^

In this study, three lung sound samples from 10 or more samples were obtained. In each institute, two or more physicians, who were licensed pediatricians, discussed the selection of sound samples without noises and with the same sound spectrum size for each individual. After deciding on the zero point, personal lung sounds were automatically calculated using an in‐house calculation software program.^9^, ^13^ We used the median values of the three samples as the measured values for each subject.

### 2.4 Statistical analyses

The statistical analyses were conducted using the SPSS software program (IBM SPSS Statistics, Version 22 for Windows; IBM Corp., Armonk, N.Y., USA). The parameters were compared using Wilcoxon's signed‐rank test. *P* values <0.05 were considered to indicate statistical significance. Bonferroni's multiple comparison test was used for multiple comparison procedure. *P* values <0.025 were considered to indicate statistical significance. The data in tables are expressed as the median and the first and the third quantile values. Fisher's exact test was used to assess independence.

## 3 RESULTS

### 3.1 The lung sound analysis

At the first visit, 398 of 443 subjects (89.8%) successfully underwent a breath sound analysis (median age: 8 months old).^14^ In this study, the parents of 268 of those 398 subjects (67.3%) filled out questionnaires when their child was 3 years old (median age: 36 months old). Eighty one of the 268 subjects (30.2%) had had an ARI within the 7 days prior to their first visit (Table 1). Table 2 shows the questionnaire results of the infants with and without an ARI in the two age groups. In this report, the zero level and the zero point were used to calculate the AUC in the sound spectrum, and all pediatricians who participated in this study agreed with this technique.

**TABLE 2:** Questionnaire results of the infants aged 3 to 12 months and 13 to 24 months of age with and without ARI

	ARI (+)		ARI (−)	
	Question (+)	Question (−)	Question (+)	Question (−)
【3‐12 months of age】				
History of wheezing, n (%)	3 ( 9.1)	30 (90.9)	14 ( 9.9)	128 (90.1)
Asthma/asthmatic bronchitis, n (%)	4 (12.2)	29 (87.8)	20 (11.4)	122 (88.6)
Allergy, n (%)	6 (18.2)	27 (81.8)	26 (18.3)	116 (81.7)
Atopic dermatitis, n (%)	5 (15.2)	28 (84.8)	16 (11.3)	126 (88.7)
History of RSV infection, n (%)	4 (12.2)	29 (87.8)	26 (18.3)	116 (81.7)
Hospitalization, n (%)	4 (12.2)	29 (87.8)	9 (6.3)	133 (93.7)
Family history of allergy, n (%)	25 (75.8)	8 (24.2)	116 (81.7)	26 (18.3)
Wheezing group, ^a^n (%)	8 (24.2)	25 (75.8)	40 (28.2)	102 (71.8)
Atopy group, ^b^n (%)	12 (36.4)	21 (63.6)	42 (29.6)	100 (70.4)
【13‐24 months of age】				
History of wheezing, n (%)	11 (22.9)	37 (77.1)	3 (6.7)	42 (93.3)
Asthma/asthmatic bronchitis, n (%)	8 (16.7)	40 (83.3)	3 (6.7)	42 (93.3)
Allergy, n (%)	6 (12.5)	42 (87.5)	4 (8.9)	41 (91.1)
Atopic dermatitis, n (%)	9 (18.8)	39 (81.2)	3 (6.7)	42 (93.3)
History of RSV infection, n (%)	8 (16.7)	40 (83.3)	3 (6.7)	42 (93.3)
Hospitalization, n (%)	5 (10.4)	43 (89.6)	1 (2.2)	44 (97.8)
Family history of allergy, n (%)	35 (72.9)	15 (27.1)	11 (24.4)	34 (65.6)
Wheezing group, ^a^n (%)	28 (58.3)	20 (41.7)	11 (24.4)	34 (65.6)
Atopy group, ^b^n (%)	11 (22.9)	37 (77.1)	4 ( 8.9)	41 (91.1)

^a^ Positive response to Questions 2, 3, or 5.

^b^ Positive response to Question 10 or 11.

To confirm any overlap between the infants with positive responses to wheezing‐related items (Question 2, 3, or 5) and those with positive responses to atopy‐related items (Question 10 or 11), we performed Fisher's exact test. The numbers of infants with both wheezing and atopy, with wheezing but without atopy, without wheezing but with atopy, and without either wheezing or atopy were 28, 41, 60, and 140, respectively. The *P* value of Fisher's exact test was 0.072.

### 3.2 Differences in the breath sound parameters in each item of questionnaire in the younger group

In the ARI‐positive group, the B_4_/A_T_ values in the infants with a history of asthma were significantly lower than those without a history of asthma or asthmatic bronchitis (Table 3). The A_3_/A_T_ and B_4_/A_T_ values in the infants with atopic dermatitis were significantly lower than those without atopic dermatitis (Table 3). The A_3_/A_T_, B_4_/A_T_, and RPF_75_ values in those with atopy were significantly lower than those without an ARI (Table 3).

**TABLE 3:** Results of the analysis of parameters in the breath sound spectrum in children with and without ARI

		ARI (+)			ARI (−)		
		Question (+)	Question (−)	*P* value	Question (+)	Question (−)	*P* value
【3‐12 months of age】							
Asthma/	A_3_/A_T_	12.1 (11.4, 12.7)^a^	14.6 (12.5, 15.8)	0.071	12.8 (11.2, 14.6)	14.8 (12.6, 16.8)	0.064
Asthmatic	B_4_/A_T_	7.5 (6.9, 7.9)	8.6 (7.9, 10.0)	**0.046**	7.9 (7.4, 9.5)	9.5 (7.4, 10.6)	0.138
Bronchitis	RPF_75_	7.3 (6.4, 8.0)	8.1 (5.8, 10.1)	0.476	7.4 (5.4, 8.5)	7.6 (6.2, 9.1)	0.315
	RPF_50_	7.2 (6.3, 7.6)	6.6 (5.6, 8.5)	0.977	6.5 (5.5, 7.8)	6.6 (5.6, 8.1)	0.563
Atopic	A_3_/A_T_	12.4 (10.6,12.5)	14.7 (12.6, 16.0)	**0.026**	14.2 (12.1,15.3)	14.4(12.6,16.8)	0.516
Dermatitis	B_4_/A_T_	7.2 (6.1, 7.5)	8.6 (7.9, 10.1)	**0.007**	8.8 (7.2, 10.0)	9.3 (7.5, 10.6)	0.333
	RPF_75_	5.5 (5.3, 6.8)	8.2 (6.5, 10.4)	0.159	7.7 (6.2, 10.7)	7.5 (6.1, 9.0)	0.297
	RPF_50_	6.9 (6.4, 7.9)	6.9 (5.6, 8.5)	0.938	7.4 (6.3, 8.9)	6.5 (5.5, 7.9)	0.112
Atopy	A_3_/A_T_	12.4 (11.6, 13.6)	15.1 (13.2, 16.4)	**0.020**	14.0 (12.0, 15.5)	14.5 (12.6, 16.9)	0.309
Group	B_4_/A_T_	7.8 (7.1, 8.4)	8.9 (8.5, 10.0)	**0.018**	8.8 (7.4, 10.1)	9.5 (7.6, 10.7)	0.184
	RPF_75_	6.4 (5.4, 7.6)*	8.5 (7.2, 10.9)**	**0.020**	7.7 (6.3, 9.5)	7.5 (6.1, 8.9)	0.194
	RPF_50_	6.6 (5.9, 7.1)	7.2 (5.3, 8.6)	0.367	7.1 (6.1, 8.6)	6.4 (5.3, 7.8)	0.059
【13‐24 months of age】							
History of wheezing	A_3_/A_T_	14.9 (13.9, 17.6)	12.8 (10.9,13.9)	**0.009**	14.4 (12.2, 16.1)	14.1 (12.2, 16.2)	1.000
	B_4_/A_T_	10.3 (8.8, 11.8)	8.1 (7.1, 9.0)	**0.006**	9.0 (8.2, 9.8)	9.1 (6.9, 10.5)	0.956
	RPF_75_	8.9 (6.0, 9.5)	7.7 (6.2, 9.6)	0.813	7.9 (5.9, 11.0)	7.7 (6.4, 8.9)	0.926
	RPF_50_	6.6 (5.0, 7.5)	6.8 (5.4, 8.9)	0.391	6.1 (5.6, 8.6)	6.2 (5.1, 9.0)	0.780

*Note*: Atopy group: infants with positive responses for atopy‐related items (Question 10 or 11).

^a^ Median (first quartile, third quartile), Bold letters represent values with a significant difference (*P* < 0.05).

^*^ *P* = 0.035, compared with the group of Question (+) and ARI (−).

^**^ *P* = 0.038, compared with the group of Question (−) and ARI (−).

Furthermore, although no significant difference was found (Bonferroni's multiple comparison test), the RPF_75_ value of the ARI‐positive and atopy‐positive infants was lower than that of the ARI‐negative and atopy‐negative infants (#; *P* = 0.038, Table 3). In contrast, the RPF_75_ value of the ARI‐positive and atopy‐negative infants was higher than that of the ARI‐negative and atopy‐negative infants (§; *P* = 0.035, Table 3).

No marked differences were observed in the other spectrum curve indices of the infants between the question‐positive and the question‐negative groups.

### 3.3 Differences in the breath sound parameters in each item of questionnaire in the older group

In the ARI‐positive group, the A_3_/A_T_ and B_4_/A_T_ values in the infants with a wheezing history were significantly higher than those without a wheezing history (*P* = 0.009 and *P* = 0.006, respectively, Table 3).

No marked differences were observed in the other spectrum curve indices of the infants between the question‐positive and question‐negative groups.

## 4 DISCUSSION

A lung sound analysis has been evaluated as a reliable, noninvasive respiratory function test.^6^, ^7^, ^9^ With recent technological advances, data collection using mobile phones^21^ and automatic analyses by artificial intelligence^22^, ^23^ have also been reported. Furthermore, the target patients have expanded to include newborn babies.^24^

In this report, we used a newly revised technique to conduct a sound spectrum analysis.^15^ Previously, to analyze the spectrum images, small differences in the power of background noises are seen among patients. These slight differences may depend on the influence of wide‐ranging outside noises and/or the difference in the pressure with which each examiner held the microphone.^15^ When the original zero level is lower than −90 dBm, the high‐pitched area may be underestimated. These factors can adversely affect the accuracy of the lung sound analysis. To resolve this problem, the zero level was visually corrected based on the lung sound spectra in each sample before the zero point (the frequency at 0 dB) was decided by at least two examiners. Using the new technique, we were able to measure the ratio of the AUC more accurately than in the past.

Through the present long‐term follow‐up study, we showed that the risk factors for asthma development were clearly associated with the results of the lung sound analysis. These findings were similar to those of two previous reports concerning first‐visit infants in the healthy period^14^ and after an ARI.^16^ Of note, in the younger group, the values of spectrum curve indices (A_3_/A_T_, B_4_/A_T_, and RPF_75_) of the infants with a history of asthma and/or asthmatic bronchitis (Question No. 7), atopic dermatitis (Question No. 11), and atopy (positive responses to Question No. 10 and/or 11) were significantly lower than those in patients without these conditions in the ARI‐positive group. In contrast, in the ARI‐negative group, there were no significant differences between them. As previous data have shown,^11^, ^25^ a decrease in the A_3_/A_T_, B_4_/A_T_ or RPF_75_ may suggest an increase in the high‐pitched area of the sound spectrum, which may indicate bronchial constriction. Our results confirm that the high‐pitched area of the spectrum curves in the infants with atopy was greater than in infants without atopy after suffering an ARI.

However, we obtained different findings in the older group, with the A_3_/A_T_ and B_4_/A_T_ values in the infants with a wheezing history being higher than those without a wheezing history in the ARI‐positive group. It is reasonable to assume that an ARI, which induces acute inflammation and/or edema of the airway mucosa, may introduce some additional sounds into the normal array of breath sounds. It has been reported that an increase in the middle‐pitched area of spectrum curves induces an increase in the spectrum curve indices.^14^ Our results suggest the possibility of an increase in the middle‐pitched area of the spectrum curves in older infants with a wheezing history who have an ARI.

Given the above findings, we believe that there may be two kinds of abnormal additional sounds related to ARIs and an atopic state: an increase in the high‐pitched area or an increase in the middle‐pitched area of the sound spectrum. These inaudible additional sounds are thought to be generated by an independent mechanism. One reason for this suspicion is that no relationships were noted between the presence of wheezing and atopy, as in the previous study.^16^ While an age‐dependent difference does appear to exist, ≤12 months of atopy‐related sounds and >12 months of infection‐related sounds, the lack of any marked difference in the spectrum curve index values in the older group may be due to the number of subjects in the older group being too small. We speculate that these differences are dependent on pure bronchial constriction (the high‐pitched sound)^9^, ^12^ and the edema and/or remodeling in the bronchial wall and peri‐wall region due to airway inflammation (the middle‐pitched sound), but we lack any supporting evidence.

Some phenotypes associated with recurrent wheezing and/or infantile asthma were reported in previous studies.^26^ Recurrent wheezing after viral infection has been noted in infants, and the pathophysiology of the virus infection‐induced recurrent wheezing/infantile asthma may be different from that of atopic asthma.^27^ Although it is often difficult to distinguish atopic asthma and infectious asthma in infants, prospective studies using palivizumab, an anti‐ respiratory syncytial virus monoclonal antibody, have also suggested that these phenotypes are independent.^28^ Our results suggest the existence of this phenotype‐dependent airway condition in early infants. We will examine this point further in future studies.

As a limitation of our study, the number of cases in some groups was small. A stratified analysis based on age should be performed because the ratio of ARI differed by patient age.^16^ This may be related to the social situation in Japan, as the ratio of children attending nursery schools clearly increases after 1 year of age, along with the frequency of respiratory tract infections. Furthermore, we were unable to indicate which part of the airway was causing these additional lung sounds.^12^ To consider the presence of pathological differences in infection‐ or atopy‐dependent additional lung sounds, our results may indicate the difference depending on the airway condition and/or the site of region. This seems to be an interesting point, and we intend to explore it further in the future.

## 5 CONCLUSIONS

Lung function tests are difficult to perform in infants who are just at the onset of asthma.^29^ In this study, we used a newly revised technique to analyze lung sounds in infants,^9^, ^15^ and this technique was proven to correspond to the lung sound analysis of infants. Our results are important because the lung sounds of infants with the risk factors for asthma development reveal residual airway changes with an ARI. A recent report proposed that a deficit in the lung function in 2‐month‐old infants suggested the development of asthma and a reduction in the airway caliber in adults.^30^

The present findings suggest that certain asthma‐related characteristics of the airway are already present in early infancy. Clinically, it is important to detect life‐long persistent abnormalities of the airways in the early infancy by using the objective method. We feel that the new criteria for asthma development in infants will be able to be established using this new technique for performing a lung sound analysis in the near future.

## FUNDING

This study was supported by the Environmental Restoration and Conservation Agency of Japan in fiscal years 2009–2017. No.1‐1.

## CONFLICT OF INTEREST

The authors have indicated they have no potential conflicts of interest to disclose.

## AUTHOR CONTRIBUTIONS

Conceptualization: Hiroyuki Mochizuki, Manabu Miyamoto

Data Collection: Manabu Miyamoto, Hiromi Shioya, Hiromi Tadaki, Tomohiko Imamura, Mayumi Enseki

Formal Analysis: Hideki Koike, Hiroyuki Furuya

Writing—Original Draft: Hiroyuki Mochizuki

Writing—Review and Editing: Shigemi Yoshihara

All authors have read and approved the final version of the manuscript.

The corresponding author or manuscript guarantor will have to confirm that he/she had full access to all of the data in the study and takes complete responsibility for the integrity of the data and the accuracy of the data analysis.

## TRANSPARENCY STATEMENT

The lead author (HM) sffirms that this manuscript is an honest, accurate, and transparent of account of the study being reported; that no important aspects of the study have been omitted; and any discrepancies from the study as planned have been explained.

## Data Availability

The data that support the findings of this study are available from the corresponding author on reasonable request.

## APPENDIX A.

**TABLE A1:** Original questionnaire (translated to English)

Child' name _____ Address _____
Sex (1, Male 2, Female), Birthday (___/___/20__), Age ___years___month, Height _______cm, Weight ____kg, Birth weight ____kg
Q1. Has your child recently caught an acute respiratory infection?
1. He/she has not had an acute respiratory infection for a week.
2. He/she recovered from an acute respiratory infection ( ) days ago.
3. He/she has an acute respiratory infection now.
Q2. When your child breathes, have you heard the sound of wheezing or whistling? (1, Yes 2, No)
Q3. When your child has had a cold, have you heard a wheezy or whistling sound? (1, Yes 2, No)
Q4. How many times has your child's chest sounded wheezy? (1, 0 times, 2, 1‐2 times, 3, 3‐6 times, 4, 7‐12 times, 5, More than 13 times)
Q5. Has your child suffered from attacks characterized by difficulty breathing with wheezing or whistling? (1, Yes 2, No)
Q6. If yes, how many such attacks has your child had? (1, 0 times, 2, 1‐2 times, 3, 3‐6 times, 4, 7‐12 times, 5, More than13 times)
Q7. Has your child been diagnosed with bronchial asthma or asthmatic bronchitis by a physician? (1, Yes 2, No)
Q8. Has your child been diagnosed with an RS virus‐induced respiratory infection? (1, Yes 2, No)
Q9. Has your child been hospitalized because of bronchial asthma, bronchitis or pneumonia? (1, Yes 2, No)
Q10. Does your child have any allergies? (1, Yes 2, No)
If yes and a blood test was performed, please select all that were positive.
(1. Mites 2. House dust 3. Cedar Pollen 4. Cat dander 5. Egg white 6. Milk 7. Others ( ))
Q11. Has your child been diagnosed with atopic dermatitis by a physician? (1, Yes 2, No)
Q12. Do your child's family have any of the allergic diseases described below?
Please connect the corresponding upper and lower words with a line.
[ Asthma, Allergic rhinitis (hay fever), Atopic dermatitis, Others]
[Father, Mother, Siblings, Grandparents]
Q13. Is there anyone who smokes in your house? Please circle all that apply. (1, Father 2, Mother 3, Others 4, None)
Q14. What kind of domestic pets do you keep? (1, Dog 2, Cat 3, Others 4, None)
Q15. Is there a road with heavy traffic near the house? (1, Yes 2, No)
